# Association between exposure to radiofrequency electromagnetic fields assessed by dosimetry and acute symptoms in children and adolescents: a population based cross-sectional study

**DOI:** 10.1186/1476-069X-9-75

**Published:** 2010-11-25

**Authors:** Sabine Heinrich, Silke Thomas, Christian Heumann, Rüdiger von Kries, Katja Radon

**Affiliations:** 1 Unit for Occupational and Environmental Epidemiology & Net Teaching, Institute and Outpatient Clinic for Occupational-, Social- and Environmental Medicine, University Hospital of Munich (LMU), Ziemssenstr. 1; 80336 Munich, Germany; 2 Department of Statistics, Ludwig-Maximilians-University, Ludwigstr. 33; 80539 Munich, Germany; 3 Institute for Social Paediatrics and Adolescent Medicine, Ludwig-Maximilians- University, Heiglhofstr. 63; 80377 Munich, Germany

## Abstract

**Background:**

The increase in numbers of mobile phone users was accompanied by some concern that exposure to radiofrequency electromagnetic fields (RF EMF) might adversely affect acute health especially in children and adolescents. The authors investigated this potential association using personal dosimeters.

**Methods:**

A 24-hour exposure profile of 1484 children and 1508 adolescents was generated in a population-based cross-sectional study in Germany between 2006 and 2008 (participation 52%). Personal interview data on socio-demographic characteristics, self-reported exposure and potential confounders were collected. Acute symptoms were assessed twice during the study day using a symptom diary.

**Results:**

Only few of the large number of investigated associations were found to be statistically significant. At noon, adolescents with a measured exposure in the highest quartile during morning hours reported a statistically significant higher intensity of headache (Odd Ratio: 1.50; 95% confidence interval: 1.03, 2.19). At bedtime, adolescents with a measured exposure in the highest quartile during afternoon hours reported a statistically significant higher intensity of irritation in the evening (4^th ^quartile 1.79; 1.23, 2.61), while children reported a statistically significant higher intensity of concentration problems (4^th ^quartile 1.55; 1.02, 2.33).

**Conclusions:**

We observed few statistically significant results which are not consistent over the two time points. Furthermore, when the 10% of the participants with the highest exposure are taken into consideration the significant results of the main analysis could not be confirmed. Based on the pattern of these results, we assume that the few observed significant associations are not causal but rather occurred by chance.

## Background

During the last decade the use of wireless communication devices has become very common in our daily life. On the other hand parts of the general population are afraid of potential negative health effects due to radiofrequency electromagnetic field (RF EMF) exposure caused by these wireless devices.

In Germany, 27% of the general population reported concerns about such potential health effects [1]. In this context, unspecific symptoms like headache, sleeping problems or difficulties in concentration were consistently attributed to self-reported mobile phone use [2,3] and to exposure to mobile phone base stations [4-7]. Most of the persons who are concerned about RF EMF exposure report symptoms to appear whilst being exposed, so acute effects on health and well-being are of special interest.

Children and adolescents are an important age group due to their high usage of mobile phones, and it is discussed if they might be more vulnerable to RF EMF. This potential for higher vulnerability is discussed due to their higher lifetime exposure (they start mobile phone use at an earlier age and use mobile phones more frequently than today's adults), their still developing nervous system and a greater conductivity of their brain tissue [8-10]. Recent studies indicated higher Specific Absorption Rate (SAR) values for children in comparison to adults [11,12], but this issue is still under discussion.

To date, only few studies investigated potential adverse health effects in young people. Two laboratory studies showed that a 900 MHz field did not have acute effects on cognitive functions in children and adolescents [13,14]. Epidemiological studies mainly investigated associations between self-reported exposure and perceived health of the participants. The results showed that self-reported mobile phone use was associated with poor perceived health [15-17]. One drawback of these studies is that the exposure assessment was based on self-reports of the participants. Earlier studies revealed that adolescents are not able to recall their mobile phone use over the past years accurately [18,19].

To avoid lack of valid exposure assessment we used personal dosimeters in our study. One advantage of these dosimeters is the possibility to assess all sources of individual exposure over the study period [20,21]. Until recently, there was only one epidemiological study in adults using personal dosimeters to assess the individual exposure, no data on children are so far available [22]. Therefore, the MobilEe study is the first epidemiological study using personal dosimetry for the assessment of exposure to RF EMF in children and adolescents, enabling objective assessment of exposure from all sources (mobile phones, DECT phones, base stations, WLAN) [23]. The aim of the study was to investigate a possible association between the individual exposure to such fields and acute health effects in children and adolescents.

## Methods

### Study design

From 2006 to 2007 children (aged 8-12 years) and adolescents (aged 13-17 years) were randomly selected from the registration offices of four Upper Bavarian (South of Germany) cities with different population sizes (Munich, Augsburg, Rosenheim, Landsberg). Informed written consent was obtained from all participants. Irrespective of their participation they were asked to fill in a short questionnaire to investigate possible differences between participants and non-participants.

Those who declared consent were invited to a local study centre where a computer-assisted personal interview (CAPI) was performed and data on participants' socio-demographic characteristics and potential confounders were collected. For children some data were collected by interviewing their parents (level of education, environmental worries).

During the 24-hour exposure measurement the participants filled in a diary recording acute symptoms at noon and in the evening before bedtime as well as the frequency of their own mobile phone use in the previous hours. Study methods have been described in detail elsewhere [23]. The study was approved by the Ethics Committee of the Medical Faculty of the Ludwig-Maximilians-University Munich (285/03).

### Exposure assessment using personal dosimetry

Exposure was measured over 24 hours using a personal dosimeter (ESM-140, Maschek Electronics, Bad Wörishofen, Germany) covering exposure to mobile phones and their base stations (GSM 900 up and down link, GSM 1800 up and down link, UMTS up and down link), to cordless phones (DECT) and to WLAN (Wireless Local Area Network) [23]. The dosimeter was placed on the upper arm of the participants opposite to the side which they usually used to hold the mobile phone during phone calls.

During night time participants were asked to fix the dosimeter next to their bed on a bottle filled with water. Due to the physical characteristics of the dosimeter valid measurements can only be obtained if the dosimeter is moved. Therefore bedtime exposure levels were not considered to be a valid proxy of night time exposure and had to be excluded from the analyses [22].

For assessing overall exposure, a combination of frequency bands had to be used due to the dosimeter's low selectivity between up- and down-link channels. Furthermore, all values below the limit of determination (0.05 V/m) were replaced by half of the limit (0.025 V/m) [23]. For each of the seven frequency bands of the dosimeter the average of the squared field strength over the relevant time period (waking hours) was taken. Each of these averages was weighted by the inverse of the respective squared ICNIRP reference levels for the electric field strength. The square root of the sum of these seven weighted averages was multiplied by 100 to obtain a percentage of the International Commission on Non-Ionizing Radiation Protection (ICNIRP) reference level [24]. The overall exposure was classified into quartiles for the main analysis [22].

### Self-reported exposure (subjective exposure)

To compare the results to findings of previous studies we also collected data on self-reported exposure. Frequency of mobile phone use during the measurement day was assessed using a diary and dichotomized for the analysis: ≤ five minutes vs. > five minutes.

### Endpoints

Using a paper-based diary, the following acute symptoms were taken into consideration and assessed twice during the 24-hour measurement (noon, in the evening before bedtime): headache, irritation, nervousness, dizziness, fatigue and concentration problems. The items were taken from the "Zerssen complaint list" [25] and assessed on a four-point Likert scale (heavy, moderate, weak, not at all). As only few participants reported heavy and moderate symptoms, the symptom variables were dichotomized. A symptom was considered present if it was reported with an at least weak intensity.

### Potential confounders

Sociodemographic data like age and level of education were collected using questions from the German Health Interview and Examination Survey for Children and Adolescents (KIGGS) [26]. Environmental worries were assessed using the short form of the Environmental Worry Scale [27]. The scale consists of 12 questions about general and specific environmental worries (e.g. noise exposure, general environmental pollution). Participants were a priori classified in "not worried" and "worried" using the median as cut-off. This was done, because no standard values exist. For children, data of their parents were used, since there is no version of the Environmental Worry Scale for children.

### Statistical analysis

Thirty measurements had to be excluded from the analysis due to technical errors. Multivariate analyses were done using logistic regression models adjusting for age, sex, level of education, study town and environmental worries stratified for children and adolescents. The potential confounding variables were defined a-priori and included in all analyses (complete-case-analyses).

We did separate analyses for the association between exposure during morning hours and the reported symptoms at noon, as well as for the association between exposure during afternoon and the reported symptoms in the evening.

In the main analysis, the potential association between measured exposure to RF EMF and acute symptoms was assessed. In a secondary analysis we used the self-reported mobile phone use of the participants during the measurement as exposure proxy.

Statistical analyses were carried out using SAS (SAS version 9.1; SAS Institute Inc., Cary, NC, USA).

## Results

### Participation and descriptive data

Overall, 1498 children and 1524 adolescents participated in the interview and the measurement (52% of those invited). Fatigue was reported most frequently especially in the evening, followed by concentration problems and headache. For all acute symptoms, except for nervousness in the evening, the prevalence was higher in adolescents than in children (Table 1).

**Table 1:** Sociodemographic data and prevalence of acute symptoms of the participating children and adolescents

	Children (n = 1484)		Adolescents (n = 1508)	
**Sociodemographic data**	**Prevalence n (%)**		**Prevalence n (%)**	

Sex: *male*	724 (48.9)		731 (48.5)	

Age: *8-10 years respectively* *13-15 years*	791 (53.3)		963 (63.9)	

Level of education at least 12 years of schooling*/grammar school^#^	939 (64.1)		771 (51.4)	

**Acute symptoms**	**Prevalence n (%)**		**Prevalence n (%)**	

	**noon**	**evening**	**noon**	**evening**

Headache	257 (17.5)	259 (17.6)	401 (27.1)	364 (24.4)

Irritation	210 (14.3)	260 (17.7)	346 (23.4)	401 (26.9)

Nervousness	173 (11.8)	225 (15.3)	188 (12.7)	226 (15.2)

Dizziness	116 (7.9)	132 (9.0)	240 (16.2)	220 (14.8)

Concentration problems	295 (20.1)	361 (24.5)	429 (29.0)	527 (35.5)

Fatigue	523 (35.7)	868 (59.1)	730 (49.4)	1058 (71.0)

* for children: parental level of education

**^# ^**for adolescents: grammar school

The overall measured exposure to RF EMF was very low and ranged from a mean of 0.13% (all measurement values below the limit of determination) to a mean of 0.92% of the ICNIRP reference level per second during waking hours (Table 2). Only 2% of the children and 14% of the adolescents used their mobile phones more than five minutes in the afternoon. The duration of mobile phone use was higher in adolescents than in children and higher in the afternoon than during morning hours (Table 2).

**Table 2:** Measured and self-reported exposure of the participating children and adolescents

	Children (n = 1484)	Adolescents (n = 1508)
**Measured exposure levels**	**% ICNIRP-reference level: mean (standard deviation); range**	

during waking hours	0.18 (0.06); 0.13 - 0.92	0.19 (0.06); 0.13 - 0.78

during morning	0.17 (0.06); 0.13 - 0.80	0.18 (0.07); 0.13 - 0.74

during afternoon	0.19 (0.08); 0.13 - 1.20	0.20 (0.07); 0.13 - 0.87

**Self-reported exposure:** **Mobile phone use**	**n (%)**	

*> 5 minutes during morning hours*	30 (2.1)	155 (10.6)

*> 5 minutes during afternoon*	34 (2.3)	212 (14.3)

### Association between self-reported and measured exposure

Measured exposure and self-reported exposure might be correlated. Therefore, we investigated if participants reporting ownership of a mobile phone and/or a long duration of use of a mobile phone have an exposure in the higher quartiles (measured exposure). Reporting a high exposure was significantly associated with the probability to have a measured exposure in quartile 2-4 in comparison to the reference category (1^st ^quartile). Nevertheless, only 25% to 30% of the children and adolescents who reported ownership of a mobile phone or longer duration of use had a measured exposure in the highest exposure quartile (Table 3).

**Table 3:** Association between self-reported and measured exposure

		Children		Adolescents	
**Variable**	**Quartile**	n (%)*	**OR (95% CI)#**	n (%)*	**OR (95% CI)#**

**Mobile phone** **ownership** yes	1	122 (18.9)	1.0	296 (23.5%)	1.0
	
	2	156 (24.2)	1.5 (1.1;2.1)	320 (25.4%)	2.1 (1.3;3.4)
	
	3	189 (29.3)	1.5 (1.3;1.8)	322 (25.5%)	2.4 (1.4;4.0)
	
	4	178 (27.6)	2.0 (1.5;2.7)	324 (25.7%)	2.6 (1.5;4.4)

**Mobile phone use** **during morning hours** > 5 minutes	1	5 (22.7)	1.0	27 (21.6)	1.0
	
	2	2 (9.1)	0.4 (0.1;2.1)	27 (21.6)	1.0 (0.6;1.8)
	
	3	9 (40.9)	1.3 (0.9;2.0)	33 (26.4)	1.2 (0.7;2.1)
	
	4	6 (27.3)	1.2 (0.4;4.1)	38 (30.4)	1.5 (0.9;2.5)

**Mobile phone use** **during afternoon hours** > 5 minutes	1	3 (10.0)	1.0	25 (13.2%)	1.0
	
	2	3 (10.0)	1.0 (0.2;5.0)	29 (15.3%)	1.2 (0.7;2.0)
	
	3	10 (33.3)	3.4 (0.9;12.6)	60 (31.6%)	2.7 (1.6;4.4)
	
	4	14 (46.4)	4.9 (1.4;17.3)	76 (40.0%)	3.6 (2.2;5.9)

95% CI 95% Confidence Interval

* Prevalence of participants with an exposure in quartile 1 -4

^# ^Odds ratio of participants with an exposure in quartile 1 -4; reference category: Quartile 1(0.13% - 0.15% of the ICNIRP reference level), OR = 1

### Association between measured exposure and acute symptoms

Children with an exposure in the 4^th ^quartile during the afternoon reported a statistically significant higher intensity of concentration problems in the evening (4^th ^quartile 1.55; 1.02- 2.33) (Table 4).

**Table 4:** Results of the logistic regression models by measured and self-reported exposure for children

Symptoms	Quartile	Prevalence N (%)	OR (95% CI)#	Prevalence N (%)	OR (95% CI)#
**Noon**	**Measured exposure during morning hours**			> 5 minutes mobile phone use during morning hours*	

Headache	1st	65 (17.6)	1.00	11 (36.7)	2.33 (0.98 -5.54)
	2nd	58 (15.9)	0.80 (0.52 -1.22)		
	3rd	63 (17.3)	1.14 (0.75 -1.73)		
	4th	71 (19.2)	1.05 (0.69 -1.62)		

Irritation	1st	54 (14.6)	1.00	6 (20.0)	0.87 (0.28 -2.68)
	2nd	51 (14.0)	0.84 (0.53 -1.34)		
	3rd	53 (14.5)	0.98 (0.62 -1.56)		
	4th	52 (14.1)	0.98 (0.61 -1.57)		

Nervousness	1st	51 (13.8)	1.00	3 (10.0)	0.44 (0.10 -1.98)
	2nd	42 (11.5)	1.47 (0.93 -2.32)		
	3rd	43 (11.8)	0.67 (0.44 -1.27)		
	4th	37 (10.0)	0.75 (0.44 -1.27)		

Dizziness	1st	25 (6.7)	1.00	2 (6.7)	0.73 (0.16 -3.32)
	2nd	28 (7.7)	0.71 (0.38 -1.34)		
	3rd	36 (9.8)	1.27 (0.71 -2.27)		
	4th	27 (7.3)	1.10 (0.60 -2.01)		

Concentration problems	1st	72 (19.5)	1.00	6 (20.0)	0.84 (0.32 -2.19)
	2nd	67 (18.4)	0.94 (0.63 -1.40)		
	3rd	75 (20.5)	1.02 (0.68 -1.53)		
	4th	81 (21.9)	0.86 (0.57 -1.30)		

Fatigue	1st	117 (31.8)	1.00	12 (40.0)	1.08 (0.50 -2.47)
	2nd	133 (36.4)	1.24 (0.89 -1.72)		
	3rd	145 (39.6)	1.18 (0.84 -1.66)		
	4th	128 (34.8)	1.36 (0.96 -1.92)		

**Evening**	**Measured exposure during afternoon hours**			> 5 minutes mobile phone use during afternoon*	

Headache	1st	65 (17.2)	1.00	11 (32.4)	1.94 (0.85 -4.42)
	2nd	66 (18.0)	0.83 (0.54 -1.28)		
	3rd	65 (17.6)	0.84 (0.53 -1.33)		
	4th	63 (17.1)	0.82 (0.51 -1.30)		

Irritation	1st	57 (15.4)	1.00	10 (30.3)	1.72 (0.76 -3.93)
	2nd	56 (15.3)	1.14 (0.73 -1.79)		
	3rd	74 (20.1)	1.19 (0.74 -1.91)		
	4th	73 (19.8)	1.39 (0.87 -2.24)		

Nervousness	1st	56 (15.1)	1.00	7 (20.6)	1.30 (0.51 -3.33)
	2nd	56 (15.3)	1.36 (0.85 -2.18)		
	3rd	55 (14.9)	1.36 (0.82 -2.24)		
	4th	58 (15.7)	1.57 (0.95 -2.62)		

Dizziness	1st	31 (8.4)	1.00	5 (15.2)	1.31 (0.42 -3.97)
	2nd	37 (10.1)	0.81 (0.45 -1.44)		
	3rd	30 (8.1)	0.73 (0.39 -1.36)		
	4th	34 (9.3)	1.06 (0.58 -1.92)		

Concentration problems	1st	76 (20.6)	1.00	13 (39.4)	1.44 (0.66 -3.16)
	2nd	92 (25.2)	1.26 (0.86 -1.86)		
	3rd	95 (25.8)	1.20 (0.79 -1.81)		
	4th	98 (25.6)	**1.55 (1.02 -2.33)**		

Fatigue	1st	211 (57.2)	1.00	18 (54.6)	0.80 (0.38 -1.70)
	2nd	215 (58.9)	0.95 (0.68 -1.32)		
	3rd	226 (61.3)	0.94 (0.66 -1.34)		
	4th	216 (59.0)	0.99 (0.69 -1.42)		

* Reference category: self-reported usage of mobile phone ≤ 5 minutes during the morning or afternoon hours

#OR = Odds ratio; 95% CI = 95% Confidence Interval; Adjusted for age, sex, level of education, study town, environmental worries

In adolescents, a significant association between measured exposure during morning hours and headache at noon (adjusted Odds Ratio 1.50; 95% CI: 1.03- 2.19 (4^th ^quartile)) as well as exposure during the afternoon and irritation in the evening (4^th ^quartile: 1.79; 1.23- 2.61) was observed (Table 5).

**Table 5:** Results of the logistic regression models by measured and self-reported exposure for adolescents

Symptoms	Quartile	Prevalence N (%)	OR (95% CI)#	Prevalence N (%)	OR (95% CI)#
**Noon**	**Measured exposure during morning hours**			> 5 minutes mobile phone use during morning hours*	

Headache	1st	89 (23.7)	1.00	54 (35.1)	**1.55 (1.05 -2.29)**
	2nd	96 (25.9)	1.08 (0.75 -1.56)		
	3rd	104 (28.3)	1.19 (0.83 -1.73)		
	4th	112 (30.5)	**1.50 (1.03 -2.19)**		

Irritation	1st	68 (18.3)	1.00	50 (32.9)	**1.64 (1.10 -2.44)**
	2nd	95 (25.5)	1.05 (0.71 -1.54)		
	3rd	86 (23.4)	1.08 (0.73 -1.59)		
	4th	97 (26.5)	1.44 (0.97 -2.15)		

Nervousness	1st	35 (9.4)	1.00	25 (16.2)	1.30 (0.79 -2.14)
	2nd	45 (12.1)	1.17 (0.72 -1.91)		
	3rd	42 (11.4)	1.13 (0.68 -1.87)		
	4th	66 (18.0)	1.39 (0.84 -2.30)		

Dizziness	1st	55 (14.7)	1.00	30 (19.5)	1.24 (0.78 -1.97)
	2nd	57 (15.4)	1.07 (0.68 -1.68)		
	3rd	58 (15.7)	1.33 (0.85 -2.07)		
	4th	70 (19.1)	1.34 (0.84 -2.11)		

Concentration	1st	99 (26.5)	1.00	49 (32.0)	1.28 (0.86 -1.89)

problems	2nd	117 (31.5)	0.99 (0.70 -1.41)		
	3rd	107 (29.0)	0.72 (0.50 -1.04)		
	4th	106 (29.0)	0.99 (0.69 -1.43)		

Fatigue	1st	156 (41.7)	1.00	90 (58.4)	**1.76 (1.22 -2.56)**
	2nd	180 (48.4)	0.98 (0.71 -1.35)		
	3rd	195 (53.4)	1.01 (0.73 -1.40)		
	4th	199 (54.2)	1.37 (0.97 -1.93)		

**Evening**	**Measured exposure during afternoon hours**			> 5 minutes mobile phone use during afternoon*	

Headache	1st	84 (22.3)	1.00	65 (30.7)	1.30 (0.92 -1.85)
	2nd	89 (23.9)	1.44 (1.00 -2.08)		
	3rd	87 (23.5)	1.04 (0.71 -1.53)		
	4th	104 (28.0)	1.39 (0.94 -2.05)		

Irritation	1st	88 (23.4)	1.00	63 (29.7)	1.14 (0.81 -1.62)
	2nd	98 (26.1)	1.17 (0.82 -1.69)		
	3rd	104 (28.0)	1.37 (0.95 -1.98)		
	4th	111 (30.0)	**1.79 (1.23 -2.61)**		

Nervousness	1st	52 (13.8)	1.00	37 (17.5)	1.18 (0.77 -1.82)
	2nd	60 (16.1)	1.15 (0.74 -1.78)		
	3rd	53 (14.4)	1.08 (0.68 -1.69)		
	4th	61 (16.5)	1.39 (0.88 -2.20)		

Dizziness	1st	48 (12.8)	1.00	32 (15.2)	0.96 (0.62 -1.51)
	2nd	55 (14.8)	1.04 (0.65 -1.65)		
	3rd	48 (13.0)	1.37 (0.87 -2.16)		
	4th	69 (18.7)	1.54 (0.96 -2.45)		

Concentration problems	1st	126 (33.7)	1.00	75 (35.4)	0.93 (0.67 -1.29)
	2nd	130 (34.9)	1.17 (0.84 -1.61)		
	3rd	136 (36.8)	1.15 (0.83 -1.60)		
	4th	135 (36.7)	1.08 (0.77 -1.53)		

Fatigue	1st	272 (72.5)	1.00	150 (70.8)	0.91 (0.64 -1.29)
	2nd	266 (71.1)	0.91 (0.64 -1.30)		
	3rd	264 (71.2)	0.91 (0.63 -1.30)		
	4th	256 (69.0)	0.93 (0.64 -1.35)		

* Reference category: self-reported usage of mobile phone ≤ 5 minutes during the morning or afternoon hours

#OR = Odds ratio; 95% CI = 95% Confidence Interval; Adjusted for age, sex, level of education, study town, environmental worries

As self-reported exposure might be correlated with the measured exposure, a multivariate analysis adjusting for these variables (self-reported mobile phone use, self-reported distance to the next mobile phone base station) was additionally carried out. However, no statistically relevant differences in the results could be seen (data not shown).

### Association between self-reported exposure and acute symptoms

No statistically significant association between the self-reported duration of mobile phone use and acute symptoms at noon or in the evening was observed for the children (Table 4). Adolescents using mobile phones more than five minutes during the morning hours reported statistically significantly more often headache (1.55; 1.05- 2.29), irritation (1.64; 1.10- 2.44) and fatigue at noon (1.8; 1.2- 2.6) (Table 5). No association was seen for exposure during the afternoon and acute symptoms in the evening.

## Discussion

The MobilEe-Study was the first study in children and adolescents using personal dosimeters to assess the individual exposure to RF EMF and to investigate a possible association between this exposure and acute symptoms.

The measured exposure levels were on average far below the current ICNIRP reference levels, which is in accordance with the results of two previous studies using personal dosimeters for exposure assessment [22,28].

Regarding a potential association between measured exposure to RF EMF and acute symptoms, some of the observed results reached the level of statistical significance. As these results were only of borderline significance and not consistent over the two time points (morning, afternoon), we believe that the observed associations are due to chance or multiple testing. Taking multiple testing into account, none of these observed associations would have reached the level of statistical significance. The results of two previous studies in adults did not show associations between RF EMF exposure and acute symptoms [29,30], either. In a sensitivity analysis exposure was also considered as a binary cut-off (cut-off 90% percentile) to compare those 10% with the highest exposure to the remaining participants (data not shown). The observed results could not confirm the significant results of the main analysis.

When self-reported mobile phone use during the measurement was taken as exposure proxy, some statistically significant results at noon were observed for the adolescents. These results are in agreement with those of another epidemiological study that revealed an association between self-reported mobile phone use and the occurrence of negative health symptoms [3]. However, it has to be kept in mind that self-reported exposure is no valid proxy for the real exposure and that it is most likely that the observed associations are due to differential misclassification based on overestimation of self-reported exposure and subjective symptoms.

Children and adolescents with high self-reported exposure did not necessarily have high measured exposure. Although most of the results showed statistically significant associations between self-reported and measured exposure, about 70% of the participants would be miss-classified if one would use the self-reported exposure as a proxy for the real exposure.

The major advantage of this study is the use of a valid exposure assessment method to assess individual exposure to RF EMF. Personal dosimetry enables accounting for all sources of exposure, considers people's mobility and is convenient to handle for study participants [20,21]. In comparison to self-reported exposure it is more accurate and less prone to possible bias.

One disadvantage of the used dosimeter was the limited selectivity to differentiate between the frequency bands, e.g. the dosimeter cannot differentiate between GSM1800, DECT and UMTS [23]. Furthermore, it was not possible to differentiate between up- and downlink channels. Another available dosimeter, the DSP-090 (Satimo, in the past Antennessa, France) has a slightly better selectivity but is not suitable for children and adolescents due to its weight and size [21].

A second problem is the fact that the body of the participant influences the measured exposure values [31]. Comparisons with free field measurements showed that personal dosimeters may underestimate real exposure [32]. The dimension of this underestimation is likely to be the same for each participant and therefore should not influence the assignment to the exposure quartiles.

Measuring the night time exposure levels is another drawback of the used dosimeter. Our study participants placed the dosimeters near their beds, which resulted in a constant, but arbitrary measurement during the night. As shown in lab measurements, valid measurements can only be obtained if the dosimeter is moved and thus, we excluded the values. Of course young people spend some hours e.g. sitting in school, but in comparison to the fixed position of the dosimeter during the night we consider that although they are sitting the arm is moved sometimes. Therefore, we assume that the daytime measurements are valid.

The participation (52%) was reasonable considering that the children and adolescents had to carry the dosimeter for 24 hours. To analyse a possible bias caused by selective non-participation, we compared those who participated in the field study to those who did not. Parents and adolescents who had a higher level of education and those who were concerned about mobile phone exposure were more likely to take part in the study (data not shown). It appears that primarily those parents and adolescents who were concerned about a possible association between RF EMF exposure and health took part in the study. We cannot rule out a preferential selection of these subjects in our study. Due to the objective exposure measurement a differential misclassification seems to be unlikely and therefore an overestimation of the results is also unlikely.

Due to the cross-sectional design of the study, exposure was only assessed for 24 hours and it might be that exposure during the study day may not be representative for a longer time period. To verify the representativeness of the measurements, 54 participants carried the dosimeter for five consecutive days. The results indicated that the exposure assessment on a single weekday reflects the typical average weekday exposure quite adequately. Between 20% and 57% of the participants were in exactly the same exposure quartile on two days of the week (perfect agreement). Highest complete agreement was always seen between two consecutive days. However, for weekdays, exposure categories differed by at most one exposure quartile for more than 80% of the population indicating that misclassification of exposure might result in an underestimation of the effect [23].

## Conclusions

In summary, we found some associations between measured exposure and acute symptoms. Due to inconsistencies between the part of the day and the fact that the results were not observed when taking the 10% of the participants with the highest exposure into consideration, we assume that these are caused by multiple testing. The results regarding self-reported exposure and symptoms are most likely due to differential misclassification and this fact emphasizes the necessity of a valid exposure assessment in epidemiological studies.

## Abbreviations

CAPI: Computer Assisted Personal Interview; DECT: Digital Enhanced Cordless Phone; RF EMF: radiofrequency electromagnetic fields; GSM: Global System for Mobile Communications; ICNIRP: International Commission on Non-Ionizing Radiation Protection; UMTS: Universal Mobile Telecommunication System; WLAN: Wireless Local Area Network.

## Competing interests

The authors declare that they have no competing interests.

## Authors' contributions

SH was one of the principle investigators responsible for design, conduct and writing the manuscript. ST was also one of the principle investigators responsible for design and acquisition of data. CH made contributions to the statistics and to draft the manuscript. RK made contributions to draft the manuscript. KR made contributions to conception and design and also to analysis and drafting the manuscript. All authors read and approved the final manuscript.
